# The Biochemical Profile of Familial Hypocalciuric Hypercalcemia and Primary Hyperparathyroidism during Pregnancy and Lactation: Two Case Reports and Review of the Literature

**DOI:** 10.1155/2016/2725486

**Published:** 2016-11-09

**Authors:** S. A. Ghaznavi, N. M. A. Saad, L. E. Donovan

**Affiliations:** ^1^Department of Medicine, Division of Endocrinology and Metabolism, University of Calgary, Calgary, AB, Canada; ^2^Department of Obstetrics and Gynaecology, University of Calgary, Calgary, AB, Canada

## Abstract

*Background*. Primary hyperparathyroidism (PHPT) and Familial Hypocalciuric Hypercalcemia (FHH) result in different maternal and fetal complications in pregnancy. Calcium to creatinine clearance ratio (CCCR) is commonly used to help distinguish these two conditions. Physiological changes in calcium handling during pregnancy and lactation can alter CCCR, making it a less useful tool to distinguish PHPT from FHH.* Cases*. A 25-year-old female presented with hypercalcemia and an inappropriately normal PTH. Her CCCR was 0.79% before pregnancy and rose to 1.99% in her second trimester. The proband's mother and neonate had asymptomatic hypercalcemia. Genetic analysis revealed a CaSR mutation consistent with FHH. A 19-year-old female presented with a history of nephrolithiasis who underwent emergent caesarean section at 29 weeks of gestation for severe preeclampsia. At delivery, she was diagnosed with hypercalcemia with an inappropriately normal PTH and a CCCR of 2.67%, which fell to 0.88% during lactation. Parathyroidectomy cured her hypercalcemia. Pathology confirmed a parathyroid adenoma.* Conclusion*. These cases illustrate the influence of pregnancy and lactation on renal calcium indices, such as the CCCR. To avoid diagnostic error of women with hypercalcemia during pregnancy and lactation, calcium biochemistry of first-degree relatives and genetic testing of select patients are recommended.

## 1. Background

Primary hyperparathyroidism (PHPT) in pregnancy is associated with hyperemesis, nephrolithiasis, pancreatitis, and preeclampsia. In the fetus, maternal PHPT can result in fetal growth restriction, severe neonatal hypocalcemia, tetany, and death [1]. However, due to the increased availability of calcium testing, the majority of patients with PHPT are now asymptomatic and appear clinically similar to patients with Familial Hypocalciuric Hypercalcemia (FHH). FHH is associated with mild to moderate degrees of hypercalcemia and does not usually cause maternal pregnancy complications. Potential fetal complications include mild hypercalcemia, severe hypocalcemia, or neonatal severe hyperparathyroidism, depending on the genotype of the fetus [1]. Parathyroidectomy is definitive therapy for patients with symptomatic PHPT but is inappropriate in those with FHH. This is because patients with FHH have a loss of function or inactivating mutation in the gene for the calcium sensing receptor (CaSR), leading to hypercalcemia starting in fetal life [2]. Although these patients have calcium levels above the reference range for the population, their degree of hypercalcemia reflects a higher set point for a physiological level of calcium; that is, they require a higher calcium level to maintain neurological, muscular, and other cellular functions. It is crucial for clinicians to differentiate sporadic PHPT from FHH in order to minimize the risk of symptomatic hypocalcemia and select the appropriate patients for parathyroidectomy.

We present two cases of women with hypercalcemia in pregnancy; patient 1 has genetically confirmed FHH, and patient 2 has histologically confirmed PHPT. The biochemistry in these two cases is summarized in Table 1. The cases reported here illustrate the effects of maternal calcium physiology on renal calcium indices including the calcium to creatinine clearance ratio (CCCR), calculated using the following formula:

(1) $$\begin{array}{c} \frac{\left[24\;\text{hr urinary calcium }\left(\text{mmol}/\text{L}\right)\times \text{serum creatinine }\left(\mu \text{mol}/\text{L}\right)\right]}{\left[\text{serum calcium }\left(\text{mmol}/\text{L}\right)\times 24\;\text{hr urinary creatinine }\left(\text{mmol}/\text{L}\right)\right]}. \end{array}$$

Due to the hypoalbuminemia of pregnancy, total serum calcium concentrations decline early in pregnancy, while ionized (physiologically active) calcium remains the same. Where available, we present albumin adjusted calcium using the following formula:

(2) Albumin  adjusted  Ca  (mmol/L)=Ca  measured  (mmol/L)+0.02∗40−Albumin  g/L.

## 2. Case Presentations

### 2.1. Patient 1

A 25-year-old Caucasian woman with documented hypercalcemia since the age of 16 presented with concerns regarding the impact of hypercalcemia on pregnancy outcomes. The patient's biochemistry prior to pregnancy showed albumin adjusted calcium of 2.61 mmol/L (reference range: 2.10–2.55 mmol/L), albumin of 44 g/L (reference range: 33–48 g/L), PTH of 33 ng/L (reference range: 10–55 ng/L), and serum phosphate of 0.99 mmol/L (reference range: 0.80–1.50 mmol/L). A 25-hydroxyvitamin D level is unknown for the patient during pregnancy, but previous 25-hydroxyvitamin D was 58.1 nmol/L (reference range: 40–110 nmol/L). Her calcium to creatinine clearance ratio (CCCR) was 0.79% prior to pregnancy, which is consistent with FHH. The proband's mother was hypercalcemic (albumin adjusted calcium = 2.74 mmol/L, albumin 36 g/L).

During the second trimester of pregnancy the patient's albumin adjusted calcium was 2.59 mmol/L, albumin was 33 g/L, and her CCCR increased to 1.99%, a value more consistent with PHPT than FHH. She had an uncomplicated delivery at 40 weeks of gestational age. A healthy male was born with asymptomatic hypercalcemia (serum calcium = 2.79 mmol/L, reference range: 1.90–2.60 mmol/L), which persisted at two years of age. While breastfeeding, the patient's albumin adjusted calcium was 2.53 mmol/L. Subsequently, the patient had two first-trimester spontaneous abortions. She then carried a fourth pregnancy to term and gave birth to a healthy female who also had asymptomatic hypercalcemia (serum calcium = 2.75 mmol/L, reference range: 1.90–2.60 mmol/L) at birth. Ultimately, genetic analysis revealed a heterozygous mutation of the* R716 CE *gene on the calcium sensing receptor and the patient was diagnosed with Familial Hypocalciuric Hypercalcemia.

### 2.2. Patient 2

A 19-year-old woman of Mexican Mennonite descent underwent an emergency caesarean delivery at 29 weeks of gestation for severe preeclampsia. She had a history of recurrent nephrolithiasis since the age of 16, but there was no prior documentation of calcium levels. Her pregnancy was complicated by calcium oxalate kidney stones and pyelonephritis and intrauterine growth restriction. Due to severe preeclampsia, the baby was delivered at 29 weeks of gestation and was found to be normocalcemic (serum calcium = 2.21 mmol/L, reference range: 1.90–2.60 mmol/L) with a birth weight of 980 grams. Blood work on her at delivery revealed albumin adjusted calcium of 2.97 mmol/L (reference range: 2.10–2.55 mmol/L), albumin of 22 g/L, a 25-hydroxyvitamin D level of 36.5 nmol/L (reference range: 80–200 nmol/L), serum phosphate of 0.66 mmol/L (reference range: 0.80–1.50 mmol/L), an inappropriately normal PTH of 52 ng/L (13–54 ng/L), and a CCCR of 2.67%. Her albumin adjusted calcium was 2.76 mmol/L, 3.18 mmol/L, and 3.11 mmol/L on days 1, 3, and 5 postpartum. Investigations repeated one month postpartum during lactation revealed albumin adjusted calcium of 2.96 mmol/L, albumin of 33 g/L, a 25-hydroxyvitamin D level of 72 nmol/L on vitamin D supplementation, serum phosphate of 0.82 mmol/L, and a PTH of 54 ng/L. Her CCCR fell to 0.89% during lactation, suggestive of FHH. Calcium biochemistry from her family was unavailable.

Given her young age at presentation, she was investigated for genetic causes of PHPT including multiple endocrine neoplasia (MEN), jaw tumor syndrome, and FHH, which were all negative.

Plans for parathyroidectomy were deferred until she could be biochemically reevaluated after she completed lactation; however, the patient became pregnant again while still breastfeeding. Her pregnancy was discovered at nine-week gestational age, at which point she was both pregnant and lactating, and her biochemistry revealed albumin adjusted calcium of 2.76 mmol/L, albumin of 38 g/L, and vitamin D level of 77.4 nmol/L. Given her prior complicated pregnancy with severe preeclampsia, fetal growth restriction, and premature delivery, second-trimester parathyroidectomy was performed resulting in normalization of serum calcium. Surgical pathology confirmed a single parathyroid adenoma weighing 174 milligrams. Despite the surgical cure, her second pregnancy was also complicated by preeclampsia and fetal growth restriction. However, she progressed 6 weeks further in her second pregnancy and delivered by caesarean section at 35 weeks of gestational age. She gave birth to a healthy normocalcemic male (serum calcium of 2.38 mmol/L, reference range: 1.90–2.60 mmol/L) with a birth weight of 1900 grams.

## 3. Discussion

### 3.1. Calcium Physiology during Pregnancy and Lactation

Maternal changes in calcium physiology occur during pregnancy in order to provide the growing fetal skeleton with adequate calcium. The dilutional hypoalbuminemia of pregnancy leads to a decline in total calcium measurements; however, the ionized calcium remains stable. Therefore, the ionized calcium is the preferred method of calcium measurement in pregnant women. A limitation of our paper is that the cases presented here were clinical cases and data has been gathered on a retrospective basis. Hence, ionized calcium measurements are not available, so albumin adjusted calcium measurements are provided.

The biochemical description of our cases is consistent with previous descriptions of “absorptive hypercalciuria” during pregnancy [3]. Absorptive hypercalciuria is a physiological change of pregnancy, defined as an elevated postprandial and 24-hour urine calcium excretion, due to increased intestinal calcium absorption of dietary calcium. In contrast, in the fasting state, the random urine calcium excretion should not be effected by absorptive hypercalciuria. The responsible physiological changes for absorptive hypercalciuria include placental production of PTHrP, a surge in the levels of 1,25 hydroxyvitamin D, and subsequent increase in the intestinal absorption of calcium and hence the increase in renal excretion of calcium [3]. In patient 1 with FHH, we saw a change in CCCR from 0.79% prepregnancy to 1.99% in the second trimester, consistent with absorptive hypercalciuria of pregnancy.

At delivery, sudden loss of placental transfer of calcium to the fetus, in addition to increased bone resorption secondary to inactivity in the postpartum period, may lead to severe hypercalcemia [4]. Women with superimposed PHPT are particularly at risk for hypercalcemic crisis in the postpartum period, especially if lactating, although this was not seen in our patient with PHPT.

Lactation is a time of relative hypocalciuria. The major regulators of calcium during lactation are breast-derived PTHrP and, to a lesser degree, low estradiol levels. Postpartum estrogen deficiency together with rising PTHrP levels increases skeletal calcium resorption and renal calcium reabsorption, which lowers the renal calcium excretion [4]. Additionally, there is a rapid fall in 1,25 hydroxyvitamin D levels during puerperium, accompanied by a decline in the intestinal absorption of calcium and a reduction in renal calcium excretion [3]. In patient 2 with PHPT, we saw a change in CCCR from 2.67% in her first pregnancy to 0.89% while lactating and vitamin D replete, consistent with relative hypocalciuria of lactation.

When interpreting renal calcium indices in pregnancy and lactation, clinicians must take into account both the physiological changes in calcium homeostasis in these states and also the superimposed pathologic changes of FHH or PHPT on calcium handling. Moreover, the relative changes in calciuria during pregnancy and lactation are not absolutes, and varying degrees of hypercalciuria and hypocalciuria may be seen in either pregnancy or lactation, further adding to the difficulty in using renal calcium indices to aid in diagnosis during the reproductive window.

## 4. Diagnosis

Determining the etiology of hypercalcemia in pregnancy is necessary for appropriate management. A high or inappropriately normal PTH level suggests PTH mediated hypercalcemia and narrows the differential to Primary Hyperparathyroidism and Familial Hypocalciuric Hypercalcemia, provided vitamin D deficiency and/or very low calcium intake, renal insufficiency, and use of thiazides or lithium have been ruled out. Clinicians employ calcium biochemistry and genetic testing to distinguish PHPT from FHH.

### 4.1. Biochemical Testing in Hypercalcemia during Pregnancy

Guidelines cite a cutoff CCCR < 1% to suggest FHH and CCCR > 2% to suggest PHPT [5]. However, there is significant overlap of renal calcium indices such as the CCCR in normal, FHH, and PHPT patients [2]. Despite this, CCCR is used in conjunction with clinical features and genetic testing when required to distinguish between FHH and PHPT outside of pregnancy. In pregnant and lactating women, changes in maternal calcium handling as well as the individual effects of PHPT or FHH on renal calcium indices make CCCR an even less useful tool to distinguish between these two conditions.

The expected relative hypercalciuria of pregnancy may lead to an elevated CCCR, resulting in misdiagnosis of FHH as PHPT and subsequent inappropriate parathyroidectomy, as was seen in a recent case series [6].

### 4.2. Genetic Testing

The gold standard for diagnosis of FHH is molecular testing of the CaSR gene, which has identified over 100 mutations that result in reduced or inactive calcium sensing receptors [7]. However, analysis of the CaSR gene has many drawbacks that should be considered prior to its use in pregnancy. Due to the heterogeneity of CaSR mutations and the limitations of current genetic testing methods, a mutation is only detected in two-thirds of cases of FHH [8]. In addition, the cost, availability, and potential time delay in obtaining results are practical limitations in the use of genetic testing to definitely diagnose FHH during pregnancy. The urgency of a diagnosis of FHH versus PHPT is far greater in pregnant women than in nonpregnant women, as the safety of a curative parathyroidectomy for PHPT is considered highest in the second trimester [9]. Testing the serum and urine calcium of three first-degree relatives results in a lower false negative rate compared to DNA sequencing of that proband, and results of biochemistry are usually available much earlier than genetic testing [10].

## 5. Pregnancy Complications

Below are the known complications of PHPT and FHH in pregnancy.

### 5.1. Maternal Complications of PHPT

PHPT in pregnancy can lead to a number of serious maternal complications including hyperemesis, nephrolithiasis, pancreatitis, preeclampsia necessitating premature delivery, and postpartum hypercalcemic crisis [1, 4]. Rates of maternal complications in PHPT are quoted as up to 67% [3]. Evidence for reduction in maternal complications after parathyroidectomy is limited and largely derived from case studies of symptomatic PHPT patients in pregnancy [11]. Some studies suggest a benefit of second-trimester parathyroidectomy, while others show a persistent risk of preeclampsia despite surgical treatment of a parathyroid adenoma up to five years prior to delivery [9, 12].

PHPT, particularly in those of reproductive age or younger, may be the first clue that an individual has multiple endocrine neoplasia 2 (MEN2). Despite the fact that MEN2 is a rare cause of PHPT, undiagnosed pheochromocytoma during pregnancy carries a high risk of maternal and fetal mortality [13]. Proper detection and treatment for pheochromocytoma brings the mortality rate down from 50% to <5% and 15%, for mother and fetus, respectively [13].

Hence, we recommend considering screening for pheochromocytoma in patients with PHPT recognized before or during pregnancy, especially if there is a raised clinical suspicion of pheochromocytoma, such as a positive family history or severe hypertension. However, it should be noted that some patients with pheochromocytoma are normotensive in pregnancy, making the diagnosis more challenging in these cases [14].

### 5.2. Fetal Complications of PHPT

Based on old reports, offspring of mothers with untreated PHPT had an 80% chance of fetal or neonatal complications including fetal growth restriction, severe neonatal hypocalcemia, tetany, and death [1]. Neonatal hypocalcemia due to fetal parathyroid gland suppression in the setting of maternal hypercalcemia is usually transient (lasting 3–5 months after birth); however, it can be delayed in onset and prolonged in some cases, and permanent hypoparathyroidism has also been described [4]. Curative parathyroidectomy in mothers with PHPT has been associated with a fourfold reduction in fetal mortality, based on a review from 1976, which included 14 out of 21 mothers with symptomatic hypercalcemia during pregnancy [11].

Rates of maternal and fetal complications in PHPT are frequently quoted as up to 67% and 80%, respectively [1]. These numbers are derived from case reports and series of PHPT at a time when expense and availability of laboratory testing limited calcium measurements to symptomatic patients [11]. More widely available calcium testing has led to the predominance of milder forms of hypercalcemia in those with PHPT. Little data is available for pregnancy outcomes in women with mild PHPT, but these individuals likely carry a lower risk of pregnancy complications than previously stated [15]. Support for this comes from a recent retrospective cohort study of registry data from 1977 to 2010 that identified over 1000 women with PHPT [16]. Compared to age-matched controls, women with PHPT had no difference in most pregnancy outcomes, including stillbirths. Unfortunately this paper did not provide information on preeclampsia. Apgar scores and anthropometric measurements were generally similar in neonates of women with and without PHPT. Calcium levels were only available on a subset of case women and therefore the degree of hypercalcemia was unknown for most of the women. The women with PHPT who later underwent parathyroidectomy, possibly indicating more severe hypercalcemia, had more episodes of stillbirth compared to those that did not undergo surgery (1.6% versus 0.2%) [16]. Although not definitive, this observation supports the hypothesis that the degree of hypercalcemia correlates directly with the risk of adverse pregnancy outcomes.

### 5.3. Maternal Complications of FHH

Most pregnant women with FHH are asymptomatic, and the condition does not typically result in pregnancy complications. Murine models predict that lactating women with superimposed FHH are at risk of hypercalcemic crisis. Inactivating mutations of the CaSR receptor in murine models has been shown to lead to an increased production of mammary PTHrP, but with decreased excretion of calcium into breast milk. The end result is increased bone resorption, lower milk calcium content, and higher serum calcium concentration [4].

### 5.4. Fetal Complications of FHH

FHH is an autosomal dominant condition; accordingly, there are three genotypic possibilities for the fetus of a mother with FHH. Both unaffected and heterozygous fetuses (with 1 inactivating mutation of the CaSR gene) are at risk for transient or permanent hypoparathyroidism due to suppression of their parathyroid glands in the setting of maternal hypercalcemia in pregnancy [4]. In severe cases, neonates may experience seizures, tetany, and death [11]. Heterozygotes may eventually develop hypercalcemia with hypocalciuria in the neonatal period. A fetus may also inherit a second CaSR mutation from the father or through a spontaneous mutation, making them homozygously dominant. This situation classically results in neonatal severe hyperparathyroidism (NSHPT) necessitating parathyroidectomy in the neonatal period [1]. In rare cases, patients with NSHPT can have a heterozygous mutation, as is described in a case report of a heterozygous neonate with NSHPT who was successfully treated with cinacalcet monotherapy [17]. Given the range of possible neonatal outcomes, it is advisable to offer genetic counseling to all pregnant women with confirmed FHH. In addition, these neonates should undergo close monitoring of calcium in the neonatal period.

## 6. Conclusions

Given the significant overlap of renal calcium indices in normal, FHH, and PHPT patients [2] and the variable effect of changes in maternal calcium handling during pregnancy and lactation on renal calcium indices, the CCCR is even more difficult to interpret during these reproductive stages than outside pregnancy.

To distinguish between FHH and PHPT in pregnancy, we recommend testing of the proband's first-degree relatives, starting with the ionized calcium (or, if unavailable, albumin adjusted calcium) and, if elevated, progressing to 24-hour urine calcium corrected for creatinine. Genetic testing of the CaSR gene should be reserved for situations when the timely biochemical testing of family members is not possible and when parathyroidectomy cannot be delayed until the patient is biochemically reevaluated after completion of lactation. While parathyroidectomy appears to reduce pregnancy complications in the setting of symptomatic and severe hypercalcemia [11], the benefit of parathyroidectomy is less clear in women with asymptomatic hypercalcemia in pregnancy secondary to mild PHPT. In women with established FHH, we recommend offering genetic counseling to assess fetal risk. Active surveillance of neonatal serum calcium is recommended for both conditions. Further research is needed to better understand the true risk of complications in mother and fetus, as well as the benefit of parathyroidectomy in the setting of mild PHPT in pregnancy.

## Figures and Tables

**Table 1:** Summary of serum and urine biochemistry in patients 1 and 2 prior to pregnancy, during pregnancy, and during lactation.

	Patient 1 (FHH)	Patient 2 (PHPT)	Reference range^*∗*^
Prior to pregnancy			

Albumin adjusted calcium (mmol/L)	2.61 mmol/L	n/a	2.10–2.55 mmol/L
PTH (ng/L)	33 ng/L	n/a	13–54 ng/L
CCCR (%)	0.79%	n/a	<2% = possible hypocalciuria <1% = hypocalciuria

Pregnancy			

Albumin adjusted calcium (mmol/L)	2.59 mmol/L	2.97 mmol/L	2.10–2.55 mmol/L
PTH (ng/L)	46 ng/L	52 ng/L	13–54 ng/L
CCCR (%)	1.99%	2.67%	n/d

Lactation			

Albumin adjusted calcium (mmol/L)	2.53 mmol/L	2.96 mmol/L	2.10–2.55 mmol/L
CCCR (%)	n/a	0.89%	n/d

n/a = not available.

n/d = not defined.

^*∗*^ = Reference ranges are for nonpregnant and nonlactating patients.

## List of Abbreviations

CaSR: Calcium sensing receptor
CCCR: Calcium to creatinine clearance ratio
FHH: Familial Hypocalciuric Hypercalcemia
MEN: Multiple endocrine neoplasia
NSHPT: Neonatal severe hyperparathyroidism
PHPT: Primary hyperparathyroidism
PTHrP: Parathyroid hormone related peptide.
